# 2,2′-(Sulfanediyldimethyl­ene)bis­(1,3-benzothia­zole)

**DOI:** 10.1107/S1600536811004478

**Published:** 2011-02-12

**Authors:** Christoph E. Strasser, Leigh-Anne de Jongh, Helgard G. Raubenheimer, Stephanie Cronje

**Affiliations:** a Department of Chemistry and Polymer Science, University of Stellenbosch, Private Bag X1, Matieland 7602, South Africa

## Abstract

In the title compound, C_16_H_12_N_2_S_3_, the two benzothia­zole groups are oriented differently with respect to the –CH_2_– groups, one being approximately staggered and one nearly eclipsed. A sulfur–π inter­action of 3.3627 (11) Å is observed between the bridging thio­ether S atom and a thia­zole ring. The crystal packing is further stabilized by inter­molecular C—H⋯N and C—H⋯π inter­actions.

## Related literature

For the preparation of the title compound, see: Rai & Braunwarth (1961 ▶). For a related structure, see: Clegg & Elsegood (2005 ▶). For S⋯π inter­actions, see: Singh *et al.* (2006 ▶).
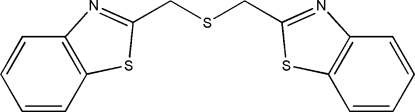


## Experimental

### 

#### Crystal data

C_16_H_12_N_2_S_3_
*M* *_r_* = 328.46
Triclinic, 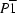

*a* = 6.3714 (10) Å
*b* = 7.8748 (13) Å
*c* = 15.339 (3) Å
α = 78.616 (3)°
β = 89.537 (3)°
γ = 74.707 (3)°
*V* = 727.0 (2) Å^3^
*Z* = 2
Mo *K*α radiation
μ = 0.50 mm^−1^
*T* = 100 K
0.15 × 0.13 × 0.13 mm

#### Data collection

Bruker APEX CCD area-detector diffractometer
Absorption correction: multi-scan (*SADABS*; Bruker, 2002 ▶) *T*_min_ = 0.809, *T*_max_ = 0.938
8237 measured reflections
2942 independent reflections
2584 reflections with *I* > 2σ(*I*)
*R*_int_ = 0.028

#### Refinement

*R*[*F* ^2^ > 2σ(*F* ^2^)] = 0.035
*wR*(*F* ^2^) = 0.088
*S* = 1.05
2942 reflections
190 parameters
H-atom parameters constrained
Δρ_max_ = 0.37 e Å^−3^
Δρ_min_ = −0.21 e Å^−3^

### 

Data collection: *SMART* (Bruker, 2002 ▶); cell refinement: *SAINT* (Bruker, 2003 ▶); data reduction: *SAINT*; program(s) used to solve structure: *SHELXS97* (Sheldrick, 2008 ▶); program(s) used to refine structure: *SHELXL97* (Sheldrick, 2008 ▶); molecular graphics: *X-SEED* (Barbour, 2001 ▶; Atwood & Barbour, 2003 ▶); software used to prepare material for publication: *X-SEED* and *PLATON* (Spek, 2009 ▶).

## Supplementary Material

Crystal structure: contains datablocks I, global. DOI: 10.1107/S1600536811004478/zq2087sup1.cif

Structure factors: contains datablocks I. DOI: 10.1107/S1600536811004478/zq2087Isup2.hkl

Additional supplementary materials:  crystallographic information; 3D view; checkCIF report

## Figures and Tables

**Table 1:** Hydrogen-bond geometry (Å, °) *Cg*1 is the centroid of the C3—C8 ring.

*D*—H⋯*A*	*D*—H	H⋯*A*	*D*⋯*A*	*D*—H⋯*A*
C1—H1*B*⋯N1^i^	0.99	2.57	3.519 (3)	161
C13—H13⋯N2^ii^	0.95	2.61	3.441 (3)	146
C1—H1*A*⋯*Cg*1^iii^	0.99	2.75	3.650 (2)	?
C9—H9*A*⋯*Cg*1^iv^	0.99	2.84	3.610 (3)	?

Symmetry codes: (i) 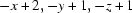; (ii) 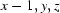; (iii) 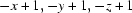; (iv) 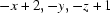.

**Fig. 1.**
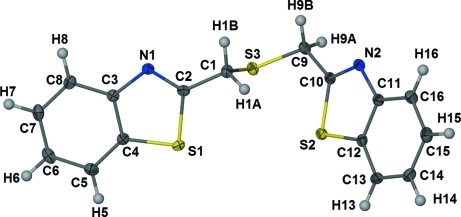
The asymmetric unit of the title compound. Ellipsoids are drawn at the 50% probability level.

## supplementary crystallographic information

### Comment

Within the benzothiazole rings of the title compound (Fig. 1) no differences are observed in bond lengths and angles. In the bridge, however, the values for the two S—C—C angles [107.72 (13)° and 113.74 (14)°] and S—C bonds [1.823 (2) and 1.803 (2) Å] differ significantly. This deviation may be explained with the two different S3—C—C—S torsion angles of 71.44 (17) and 27.1 (2)° of the respective benzothiazolyl moieties.

A related structure where one benzothiazole and one benzimidazole moiety is present was determined by Clegg & Elsegood (2005). The arrangement of the molecule in this structure is similar to the one determined here, albeit the orientation of the heterocycles with respect to the CH_2_ groups is more uniform [values for the S1—C9—C10—N3(H) and S1—C1—C2—S2 torsion angles -90.6 (7)° and -69.4 (6)°, respectively].

Interactions between neighbouring molecules in the title compound are summarized in Table 1. They include two unique C—H···N contacts between H1B and N1^i^ [symmetry code (i) –*x* + 2, –*y* + 1, –*z* + 1] as well as between H13 and N2^ii^ [symmetry code (ii) *x* – 1, *y*, *z*], leading to columns of associated molecules running parallel to the crystallographic *a* axis. The hydrogen atoms H1A and H9A of the CH_2_ groups are furthermore engaged in C—H···π interactions with benzo groups at distances of 2.75 Å [H1A···*Cg*1^iii^, (iii) –*x* + 1, –*y* + 1, –*z* + 1] and 2.84 Å [H9A···*Cg*1^iv^, (iv) –*x* + 2, –*y*, –*z* + 1] [C1···*Cg*1^iii^ 3.650 (2) Å, C9···*Cg*1^iv^ 3.610 (3) Å].

An offset parallel thiazole ring stacking [centroid distance *Cg*2···*Cg*2^iii^ 3.6724 (12) Å] of the rings containing S1 related by a centre of inversion is also found. Finally, a S···π interaction is observed between S3 and the thiazole ring containing S1, the S3···*Cg*2^iv^ distance is 3.3627 (11) Å which is shorter than the thione S···π interaction (3.631 Å) found in the solid state of 5-(2-chloroethyl)-6-methyl-2-thiouracil (Singh *et al.*, 2006).

### Experimental

The compound was synthesized from 2,2'-thiodiethanoic acid and *o*-aminothiophenol as described in the literature procedure for the preparation of 2,2'-[thiobis(ethylene)]bis(benzo-1,3-thiazole) (Rai & Braunwarth, 1961).

NMR (CD_2_Cl_2_): ^1^H (400 MHz): *δ* 7.95 (m, 2 H), 7.87 (m, 2 H), 7.47 (m, 2 H), 7.38 (m, 2 H), 4.24 (s, 4 H, CH_2_) p.p.m.; ^13^C{^1^H} (101 MHz): *δ* 169.0, 153.8, 136.4, 126.6, 125.7, 123.5, 122.2, 34.4 p.p.m.

MS (ESI): *m*/*z* (intensity) 332.0331 (2%, C_15_^13^CH_13_N_2_S_2_^34^S^+^ calcd. 332.0227), 331.0282 (15, C_16_H_13_N_2_S_2_^34^S^+^ calcd. 331.0193), 330.0335 (18, C_15_^13^CH_13_N_2_S_3_^+^ calcd. 330.0270), 329.0242 (100, C_16_H_13_N_2_S_3_^+^ calcd. 329.0235), 295.0449 (4, C_16_H_11_N_2_S_2_^+^ calcd. 295.0358).

### Refinement

All H atoms were positioned geometrically (C—H = 0.95 Å for aromatic CH and 0.99 Å for CH_2_ groups, respectively) and constrained to ride on their parent atoms with *U*_iso_(H) values set at 1.2 times *U*_eq_(C).

### Crystal data

**Table:**

C_16_H_12_N_2_S_3_	*Z* = 2
*M**_r_* = 328.46	*F*(000) = 340
Triclinic, *P*1	*D*_x_ = 1.500 Mg m^−^^3^
Hall symbol: -P 1	Mo *K*α radiation, λ = 0.71073 Å
*a* = 6.3714 (10) Å	Cell parameters from 2489 reflections
*b* = 7.8748 (13) Å	θ = 2.7–26.3°
*c* = 15.339 (3) Å	µ = 0.50 mm^−^^1^
α = 78.616 (3)°	*T* = 100 K
β = 89.537 (3)°	Prism, colourless
γ = 74.707 (3)°	0.15 × 0.13 × 0.13 mm
*V* = 727.0 (2) Å^3^	

### Data collection

**Table:**

Bruker APEX CCD area-detector diffractometer	2942 independent reflections
Radiation source: fine-focus sealed tube	2584 reflections with *I* > 2σ(*I*)
graphite	*R*_int_ = 0.028
ω scans	θ_max_ = 26.4°, θ_min_ = 2.7°
Absorption correction: multi-scan (*SADABS*; Bruker, 2002)	*h* = −7→7
*T*_min_ = 0.809, *T*_max_ = 0.938	*k* = −9→9
8237 measured reflections	*l* = −19→19

### Refinement

**Table:**

Refinement on *F*^2^	Primary atom site location: structure-invariant direct methods
Least-squares matrix: full	Secondary atom site location: difference Fourier map
*R*[*F*^2^ > 2σ(*F*^2^)] = 0.035	Hydrogen site location: inferred from neighbouring sites
*wR*(*F*^2^) = 0.088	H-atom parameters constrained
*S* = 1.05	*w* = 1/[σ^2^(*F*_o_^2^) + (0.0441*P*)^2^ + 0.3246*P*] where *P* = (*F*_o_^2^ + 2*F*_c_^2^)/3
2942 reflections	(Δ/σ)_max_ = 0.001
190 parameters	Δρ_max_ = 0.37 e Å^−^^3^
0 restraints	Δρ_min_ = −0.21 e Å^−^^3^

### Special details

**Table:**

Geometry. All e.s.d.'s (except the e.s.d. in the dihedral angle between two l.s. planes) are estimated using the full covariance matrix. The cell e.s.d.'s are taken into account individually in the estimation of e.s.d.'s in distances, angles and torsion angles; correlations between e.s.d.'s in cell parameters are only used when they are defined by crystal symmetry. An approximate (isotropic) treatment of cell e.s.d.'s is used for estimating e.s.d.'s involving l.s. planes.
Refinement. Refinement of *F*^2^ against ALL reflections. The weighted *R*-factor *wR* and goodness of fit *S* are based on *F*^2^, conventional *R*-factors *R* are based on *F*, with *F* set to zero for negative *F*^2^. The threshold expression of *F*^2^ > 2σ(*F*^2^) is used only for calculating *R*-factors(gt) *etc*. and is not relevant to the choice of reflections for refinement. *R*-factors based on *F*^2^ are statistically about twice as large as those based on *F*, and *R*- factors based on ALL data will be even larger.

### Fractional atomic coordinates and isotropic or equivalent isotropic displacement parameters (Å^2^)

**Table:**

	*x*	*y*	*z*	*U*_iso_*/*U*_eq_	
S1	0.53975 (8)	0.21565 (6)	0.51882 (3)	0.01656 (13)	
N1	0.7847 (3)	0.3908 (2)	0.42435 (10)	0.0151 (3)	
C1	0.9069 (3)	0.2967 (3)	0.58191 (13)	0.0172 (4)	
H1A	0.8209	0.3306	0.6328	0.021*	
H1B	1.0025	0.3779	0.5661	0.021*	
S2	0.82962 (8)	0.06186 (7)	0.79611 (3)	0.01754 (14)	
N2	1.1321 (3)	0.1947 (2)	0.84450 (11)	0.0168 (4)	
C2	0.7589 (3)	0.3120 (2)	0.50442 (13)	0.0146 (4)	
S3	1.07030 (8)	0.06391 (6)	0.61145 (3)	0.01686 (13)	
C3	0.6238 (3)	0.3805 (2)	0.36660 (13)	0.0154 (4)	
C4	0.4736 (3)	0.2891 (2)	0.40572 (12)	0.0155 (4)	
C5	0.3029 (3)	0.2694 (3)	0.35627 (14)	0.0192 (4)	
H5	0.2021	0.2076	0.3834	0.023*	
C6	0.2851 (3)	0.3430 (3)	0.26593 (14)	0.0222 (5)	
H6	0.1697	0.3317	0.2306	0.027*	
C7	0.4337 (3)	0.4336 (3)	0.22579 (13)	0.0207 (4)	
H7	0.4178	0.4823	0.1637	0.025*	
C8	0.6032 (3)	0.4535 (3)	0.27507 (13)	0.0190 (4)	
H8	0.7035	0.5153	0.2475	0.023*	
C9	1.2192 (3)	0.0752 (3)	0.70864 (13)	0.0189 (4)	
H9A	1.3282	−0.0417	0.7291	0.023*	
H9B	1.2992	0.1681	0.6919	0.023*	
C10	1.0779 (3)	0.1183 (3)	0.78436 (12)	0.0161 (4)	
C11	0.9748 (3)	0.2116 (3)	0.90792 (13)	0.0163 (4)	
C12	0.7962 (3)	0.1475 (3)	0.89280 (12)	0.0160 (4)	
C13	0.6216 (3)	0.1623 (3)	0.94854 (13)	0.0197 (4)	
H13	0.4992	0.1214	0.9367	0.024*	
C14	0.6321 (3)	0.2384 (3)	1.02160 (13)	0.0212 (4)	
H14	0.5158	0.2489	1.0610	0.025*	
C15	0.8112 (3)	0.3003 (3)	1.03843 (13)	0.0218 (4)	
H15	0.8152	0.3514	1.0893	0.026*	
C16	0.9823 (3)	0.2883 (3)	0.98216 (13)	0.0201 (4)	
H16	1.1031	0.3314	0.9937	0.024*	

### Atomic displacement parameters (Å^2^)

**Table:**

	*U*^11^	*U*^22^	*U*^33^	*U*^12^	*U*^13^	*U*^23^
S1	0.0170 (3)	0.0169 (3)	0.0167 (2)	−0.0062 (2)	0.00418 (18)	−0.00351 (19)
N1	0.0156 (8)	0.0127 (8)	0.0178 (8)	−0.0045 (7)	0.0012 (6)	−0.0040 (6)
C1	0.0226 (10)	0.0131 (9)	0.0165 (10)	−0.0057 (8)	−0.0003 (8)	−0.0034 (8)
S2	0.0194 (3)	0.0194 (3)	0.0163 (2)	−0.0091 (2)	−0.00021 (19)	−0.00424 (19)
N2	0.0154 (8)	0.0172 (8)	0.0172 (8)	−0.0037 (7)	−0.0014 (6)	−0.0029 (7)
C2	0.0154 (9)	0.0108 (9)	0.0188 (10)	−0.0038 (8)	0.0024 (7)	−0.0058 (7)
S3	0.0175 (3)	0.0159 (3)	0.0170 (3)	−0.00225 (19)	−0.00110 (19)	−0.00597 (19)
C3	0.0156 (10)	0.0105 (9)	0.0190 (10)	−0.0010 (8)	0.0006 (8)	−0.0042 (7)
C4	0.0157 (10)	0.0129 (9)	0.0170 (9)	−0.0016 (8)	0.0025 (7)	−0.0042 (8)
C5	0.0143 (10)	0.0152 (10)	0.0271 (11)	−0.0013 (8)	0.0010 (8)	−0.0053 (8)
C6	0.0218 (11)	0.0158 (10)	0.0278 (11)	0.0002 (8)	−0.0069 (9)	−0.0084 (9)
C7	0.0260 (11)	0.0144 (10)	0.0181 (10)	0.0009 (8)	−0.0047 (8)	−0.0027 (8)
C8	0.0229 (11)	0.0151 (10)	0.0180 (10)	−0.0040 (8)	0.0023 (8)	−0.0029 (8)
C9	0.0158 (10)	0.0230 (11)	0.0177 (10)	−0.0032 (8)	−0.0012 (8)	−0.0065 (8)
C10	0.0156 (10)	0.0139 (9)	0.0169 (10)	−0.0029 (8)	−0.0029 (8)	0.0001 (8)
C11	0.0158 (10)	0.0130 (9)	0.0177 (10)	−0.0023 (8)	−0.0022 (8)	0.0007 (8)
C12	0.0195 (10)	0.0129 (9)	0.0150 (9)	−0.0051 (8)	−0.0022 (8)	−0.0003 (7)
C13	0.0191 (10)	0.0201 (10)	0.0197 (10)	−0.0086 (8)	0.0006 (8)	0.0006 (8)
C14	0.0211 (11)	0.0219 (11)	0.0187 (10)	−0.0042 (9)	0.0039 (8)	−0.0016 (8)
C15	0.0250 (11)	0.0212 (11)	0.0184 (10)	−0.0030 (9)	−0.0013 (8)	−0.0067 (8)
C16	0.0205 (10)	0.0192 (10)	0.0219 (10)	−0.0061 (9)	−0.0024 (8)	−0.0064 (8)

### Geometric parameters (Å, °)

**Table:**

S1—C4	1.7346 (19)	C6—C7	1.399 (3)
S1—C2	1.7503 (19)	C6—H6	0.9500
N1—C2	1.292 (2)	C7—C8	1.383 (3)
N1—C3	1.389 (2)	C7—H7	0.9500
C1—C2	1.489 (3)	C8—H8	0.9500
C1—S3	1.823 (2)	C9—C10	1.501 (3)
C1—H1A	0.9900	C9—H9A	0.9900
C1—H1B	0.9900	C9—H9B	0.9900
S2—C12	1.736 (2)	C11—C16	1.397 (3)
S2—C10	1.751 (2)	C11—C12	1.399 (3)
N2—C10	1.291 (3)	C12—C13	1.392 (3)
N2—C11	1.390 (2)	C13—C14	1.381 (3)
S3—C9	1.803 (2)	C13—H13	0.9500
C3—C8	1.401 (3)	C14—C15	1.398 (3)
C3—C4	1.406 (3)	C14—H14	0.9500
C4—C5	1.390 (3)	C15—C16	1.380 (3)
C5—C6	1.386 (3)	C15—H15	0.9500
C5—H5	0.9500	C16—H16	0.9500
			
C4—S1—C2	88.91 (9)	C7—C8—H8	120.7
C2—N1—C3	110.15 (16)	C3—C8—H8	120.7
C2—C1—S3	107.72 (13)	C10—C9—S3	113.74 (14)
C2—C1—H1A	110.2	C10—C9—H9A	108.8
S3—C1—H1A	110.2	S3—C9—H9A	108.8
C2—C1—H1B	110.2	C10—C9—H9B	108.8
S3—C1—H1B	110.2	S3—C9—H9B	108.8
H1A—C1—H1B	108.5	H9A—C9—H9B	107.7
C12—S2—C10	88.55 (9)	N2—C10—C9	122.79 (18)
C10—N2—C11	110.20 (17)	N2—C10—S2	116.52 (15)
N1—C2—C1	123.58 (17)	C9—C10—S2	120.68 (15)
N1—C2—S1	116.45 (15)	N2—C11—C16	125.20 (18)
C1—C2—S1	119.96 (14)	N2—C11—C12	115.28 (17)
C9—S3—C1	99.65 (9)	C16—C11—C12	119.52 (18)
N1—C3—C8	124.87 (17)	C13—C12—C11	121.76 (18)
N1—C3—C4	115.51 (17)	C13—C12—S2	128.76 (16)
C8—C3—C4	119.62 (18)	C11—C12—S2	109.43 (14)
C5—C4—C3	121.90 (18)	C14—C13—C12	117.83 (19)
C5—C4—S1	129.11 (15)	C14—C13—H13	121.1
C3—C4—S1	108.99 (14)	C12—C13—H13	121.1
C6—C5—C4	117.59 (18)	C13—C14—C15	121.05 (19)
C6—C5—H5	121.2	C13—C14—H14	119.5
C4—C5—H5	121.2	C15—C14—H14	119.5
C5—C6—C7	121.29 (19)	C16—C15—C14	120.97 (19)
C5—C6—H6	119.4	C16—C15—H15	119.5
C7—C6—H6	119.4	C14—C15—H15	119.5
C8—C7—C6	121.10 (19)	C15—C16—C11	118.84 (19)
C8—C7—H7	119.5	C15—C16—H16	120.6
C6—C7—H7	119.5	C11—C16—H16	120.6
C7—C8—C3	118.51 (18)		
			
C3—N1—C2—C1	178.89 (17)	C1—S3—C9—C10	64.24 (16)
C3—N1—C2—S1	0.4 (2)	C11—N2—C10—C9	−177.69 (17)
S3—C1—C2—N1	−106.97 (19)	C11—N2—C10—S2	1.1 (2)
S3—C1—C2—S1	71.44 (17)	S3—C9—C10—N2	−154.18 (16)
C4—S1—C2—N1	−0.29 (16)	S3—C9—C10—S2	27.1 (2)
C4—S1—C2—C1	−178.80 (16)	C12—S2—C10—N2	−0.75 (16)
C2—C1—S3—C9	−176.45 (13)	C12—S2—C10—C9	178.10 (16)
C2—N1—C3—C8	179.46 (18)	C10—N2—C11—C16	179.98 (19)
C2—N1—C3—C4	−0.4 (2)	C10—N2—C11—C12	−1.0 (2)
N1—C3—C4—C5	179.49 (17)	N2—C11—C12—C13	−177.23 (17)
C8—C3—C4—C5	−0.4 (3)	C16—C11—C12—C13	1.8 (3)
N1—C3—C4—S1	0.2 (2)	N2—C11—C12—S2	0.5 (2)
C8—C3—C4—S1	−179.68 (15)	C16—C11—C12—S2	179.54 (15)
C2—S1—C4—C5	−179.21 (19)	C10—S2—C12—C13	177.61 (19)
C2—S1—C4—C3	0.05 (14)	C10—S2—C12—C11	0.12 (14)
C3—C4—C5—C6	0.2 (3)	C11—C12—C13—C14	−1.8 (3)
S1—C4—C5—C6	179.35 (15)	S2—C12—C13—C14	−179.05 (16)
C4—C5—C6—C7	0.1 (3)	C12—C13—C14—C15	0.7 (3)
C5—C6—C7—C8	−0.3 (3)	C13—C14—C15—C16	0.4 (3)
C6—C7—C8—C3	0.1 (3)	C14—C15—C16—C11	−0.5 (3)
N1—C3—C8—C7	−179.63 (17)	N2—C11—C16—C15	178.31 (18)
C4—C3—C8—C7	0.2 (3)	C12—C11—C16—C15	−0.7 (3)

### Hydrogen-bond geometry (Å, °)

**Table:**

Cg1 is the centroid of the C3—C8 ring.

**Table:**

*D*—H···*A*	*D*—H	H···*A*	*D*···*A*	*D*—H···*A*
C1—H1B···N1^i^	0.99	2.57	3.519 (3)	161
C13—H13···N2^ii^	0.95	2.61	3.441 (3)	146
C1—H1A···*Cg*1^iii^	0.99	2.75	3.650 (2)	?
C9—H9A···*Cg*1^iv^	0.99	2.84	3.610 (3)	?

Symmetry codes: (i) −*x*+2, −*y*+1, −*z*+1; (ii) *x*−1, *y*, *z*; (iii) −*x*+1, −*y*+1, −*z*+1; (iv) −*x*+2, −*y*, −*z*+1.

## References

[bb1] Atwood, J. L. & Barbour, L. J. (2003). *Cryst. Growth Des.* **3**, 3–8.

[bb2] Barbour, L. J. (2001). *J. Supramol. Chem.* **1**, 189–191.

[bb3] Bruker (2002). *SADABS* and *SMART.* Bruker AXS Inc., Madison, Wisconsin, USA.

[bb4] Bruker (2003). *SAINT.* Bruker AXS Inc., Madison, Wisconsin, USA.

[bb5] Clegg, W. & Elsegood, M. R. J. (2005). Private communication (refcode: SAWTOA). CCDC, Cambridge, England.

[bb6] Rai, C. & Braunwarth, J. B. (1961). *J. Org. Chem.* **26**, 3434–3436.

[bb7] Sheldrick, G. M. (2008). *Acta Cryst.* A**64**, 112–122. 10.1107/S0108767307043930 18156677

[bb8] Singh, F. V., Kumar, R., Sharon, A., Broder, C. K., Howard, J. A. K., Goel, A. & Maulik, P. R. (2006). *J. Mol. Struct.* **782**, 55–59.

[bb9] Spek, A. L. (2009). *Acta Cryst.* D**65**, 148–155. 10.1107/S090744490804362X PMC2631630 19171970

